# Cruel Treatment of a Female Patient by Her Parents

**Published:** 1887-10-01

**Authors:** 


### CRUEL TREATMENT OF A FEMALE PATIENT BY HER PARENTS.

With reference to the alleged cruel treatment of a lunatic by her parents, and the subsequent removal of the patient to a county asylum (16 and 17 Vict., cap. 97, sec. 68), to which we referred previously (*vide* vol. ii, p. 322), we are requested by the mother to insert the following letter :—

"A magisterial inquiry was held at Rugby on the 16th of Feb. 1886. I, who am surely the chief witness of all that took place in our own house, was working in my district at the time, and received no summons to attend this inquiry. How could I refute the doctor's evidence, which he states in the second inquiry to be hearsay ?"

We insert the above paragraph relative to the placing of a patient in a county asylum under the Act ; but we are certain that the Visiting Justices never detain such cases against the wishes of their friends without the most valid reasons, and it is manifestly to the advantage of the medical superintendent to have the fewest possible number of patients under his care. The safety of the patient would therefore seem to be the only reason for the detention of the case. We understand that the lunatic is paid for as a private patient. If this be so, the father should apply for the discharge of his daughter, at the same time entering into an undertaking to place her at once in a private asylum. If not successful in his application there are two courses open—first, to demand a jury ; and second, to apply for a writ of *habeas corpus*. It is possible that neither of these might tend to the fulfilment of the wishes of the mother, but either would ensure a perfectly impartial hearing.

